# Quercetin as a Candidate Adjunct for Calcineurin Inhibitor-Induced Adverse Effects in Transplant Patients

**DOI:** 10.34172/apb.025.46462

**Published:** 2025-12-22

**Authors:** Zahra Nazari Taloki

**Affiliations:** ^1^Cellular and Molecular Biology Research Center, Health Research Institute, Babol University of Medical Sciences, Babol, Iran; ^2^Department of Clinical Pharmacy, School of Pharmacy, Babol University of Medical Sciences, Babol, Iran

## To Editor,

 Calcineurin inhibitors (CNIs), including cyclosporine and tacrolimus, have been cornerstone immunosuppressants for nearly five decades, significantly improving organ transplant outcomes and patient survival. Nevertheless, their use is frequently associated with adverse effects such as organ toxicities—predominantly nephrotoxicity, cardiotoxicity, hepatotoxicity, and neurotoxicity—and metabolic syndrome, which significantly impair post-transplant quality of life.^1^

 Given the critical need to mitigate these adverse effects, we propose quercetin as a promising adjunctive agent in CNI-based immunosuppressive protocols. Quercetin is a naturally abundant flavonoid extensively studied for its multifaceted pharmacological activities, including potent antioxidant, anti-inflammatory, metabolic regulatory, and cytoprotective properties^2^, supported by a favorable safety profile in clinical use.^3^ Molecular docking and biochemical studies have demonstrated that quercetin can directly interact with calcineurin’s active site and inhibit its phosphatase activity, likely by inducing conformational changes.^4,5^ While this mechanism shares a common target with cyclosporine and tacrolimus, the inhibitory potency and selectivity of quercetin are distinct and not directly comparable to those of the clinically used drugs.Nonetheless, this finding warrants further investigation into its potential role as a complementary agent, through which it may contribute to immunosuppressive effects. Moreover, quercetin may modulate drug-metabolizing enzymes and transporters, altering systemic exposure to calcineurin inhibitors and thereby reducing toxicity. This possibility requires careful evaluation in controlled studies that consider both the risk of excessive immunosuppression and graft rejection.^6^ Quercetin’s protective effects on the kidneys, heart, and liver have been extensively demonstrated in experimental models of CNI-induced toxicity. Its antioxidant properties attenuate oxidative stress by neutralizing reactive oxygen species and enhancing the activity of endogenous antioxidant enzymes such as superoxide dismutase and glutathione peroxidase. Furthermore, quercetin and its precursor, kaempferol alleviate inflammation by suppressing pro-inflammatory cytokines (TNF-α, IL-6) and inhibiting the NF-kB signaling pathway, both of which contribute to CNI-induced tissue damage.^7-9^ The neuroprotective actions of quercetin are equally noteworthy. It activates the NRF2-ARE pathway, enhances cellular antioxidant defenses, and promotes autophagy and mitochondrial biogenesis through sirtuin-1 (SIRT1) activation.^10^ Quercetin’s phytoestrogenic effects further support neuronal survival and synaptic plasticity, potentially counteracting CNI-induced neurotoxicity and cognitive impairments frequently reported in transplant patients. However, no direct clinical studies have yet evaluated quercetin’s effects specifically in transplant populations receiving CNIs. Quercetin also exerts notable metabolic benefits, including the regulation of blood pressure via endothelial nitric oxide synthase (eNOS) activation, improvement of insulin sensitivity through AMPK activation, inhibition of intestinal carbohydrate absorption by blocking α-glucosidase, and modulation of adipokines profiles by increasing adiponectin and decreasing leptin levels. Additionally, it inhibits xanthine oxidase and reduces uric acid production, collectively addressing multiple components of post-transplant metabolic syndrome—such as hypertension, hyperglycemia, dyslipidemia, and hyperuricemia—commonly observed in recipients undergoing CNI therapy.^11-13^

 The accumulating preclinical and clinical evidence positions quercetin as a promising multi-targeted nutraceutical capable of enhancing the safety profile of calcineurin inhibitors and improving overall transplant outcomes. Well-designed prospective clinical trials are warranted to establish its optimal dosage, safety, and long-term efficacy when used alongside CNIs. However, given the potential for pharmacokinetic interactions that may reduce systemic CNI exposure, the co-administration of quercetin should currently be limited to rigorously monitored clinical settings with strict therapeutic drug monitoring. We commend your journal for its pioneering role in advancing research in this innovative field.

## Competing Interests

 The author has no potential conflicts of interest to disclose.

## Ethical Approval

 Not applicable.
